# Synthesis of Waterborne Polyurethane Using Phosphorus-Modified Rigid Polyol and its Physical Properties

**DOI:** 10.3390/polym13030432

**Published:** 2021-01-29

**Authors:** Taewoo Jang, Hye Jin Kim, Jeong Beom Jang, Tae Hee Kim, Wonjoo Lee, Bongkuk Seo, Weon Bae Ko, Choong-Sun Lim

**Affiliations:** 1 Research Center for Advanced Specialty Chemicals, Korea Research Institute of Chemical Technology, 45, Jongga-ro, Yugok-dong, Jung-gu, Ulsan 44412, Korea; wkdxodn0@krict.re.kr (T.J.); hyejink@krict.re.kr (H.J.K.); beom3374@krict.re.kr (J.B.J.); kimth@krict.re.kr (T.H.K.); winston@krict.re.kr (W.L.); bksea@krict.re.kr (B.S.); 2 Department of Convergence Science, Graduate School, Sahmyook University, Seoul 01795, Korea

**Keywords:** polyurethane, phosphorous-containing polyol, waterborne polyurethane, thermal stability

## Abstract

In this study, a phosphorous-containing polyol (P-polyol) was synthesized and reacted with isophorone diisocyanate (IPDI) to produce water-dispersed polyurethane. To synthesize waterborne polyurethanes (WPUs), mixtures of P-polyol and polycarbonate diol (PCD) were reacted with IPDI, followed by the addition of dimethylol propionic acid, to confer hydrophilicity to the produced polyurethane. An excess amount of water was used to disperse polyurethane in water, and the terminal isocyanate groups of the resulting WPUs were capped with ethylene diamine. P-polyol:PCD molar ratios of 0.1:0.9, 0.2:0.8, and 0.3:0.7 were used to synthesize WPUs. The films prepared by casting and drying the synthesized WPUs in plastic Petri dishes were used to test the changes in physical properties induced by changing the P-polyol:PCD molar ratio. The experimental results revealed that the tensile strength of PU-10, the WPU with a P-polyol:PCD molar ratio of 0.1:0.9, was 16% higher than that of the reference P-polyol–free WPU sample. Moreover, the thermal decomposition temperature of PU-10 was 27 °C higher than that of the reference sample.

## 1. Introduction

Polyurethane, which is a multipurpose polymer with good chemical resistance and high thermal and mechanical properties, is used for industrial applications, such as coatings, adhesives, textiles, and paints, and in the automotive industry [1,2,3]. However, the use of volatile organic compound-dissolving polymers causes environmental problems [4]. Therefore, eco-friendly water-dispersed polyurethane adhesives or coatings are required for the surface treatment of leather, paper, or plastics [5,6,7,8,9]. The hydrophilicity of waterborne polyurethanes (WPUs) is different from that of elastomeric polyurethane because WPUs contain hydrophilic polyols, such as dimethylol propionic acid (DMPA). Although hydrophilic polyols plays an important role in the dispersibility of WPUs in water, polyols decrease the water resistance and storage stability of WPUs [10,11,12,13,14]. These drawbacks can be compensated by adding cationic or anionic functional groups to the main polyol backbone of polyurethane [15,16]. However, the poor mechanical properties and low thermal stability of WPUs are still shortcomings for their practical applications [17,18]. Therefore, the modification of the physical properties of WPUs via the addition of nanofillers to WPU solutions to form robust composites [19,20] or via the incorporation of functional materials as chain extenders during WPU preparation has been widely studied [21].

**Figure 1:**
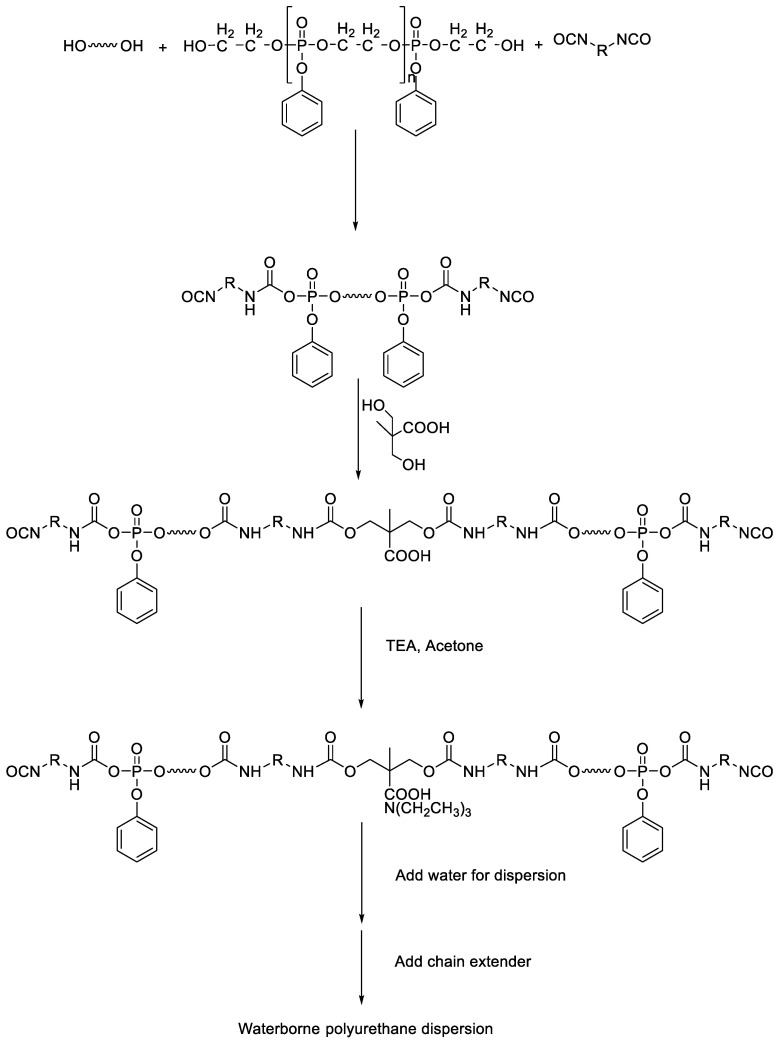
Schematic diagram of waterborne polyurethane synthesis; triethylamine (TEA).

We previously synthesized an aromatic group-rich phosphorous-containing polyol (P-polyol) by reacting ethylene glycol with phenylphosphonic dichloride, followed by the addition of epoxy to prepare elastomeric polyurethane and determined that the storage modulus of elastomeric polyurethane was 39% higher than that of P-polyol. [22] In this study, we synthesized WPU with remarkable mechanical and thermal properties by reacting mixtures of different amounts of P-polyol and polycarbonate diol (PCD) with isophorone diisocyanate (IPDI) (Figure 1). The mechanical properties of the obtained WPU films were measured using a universal testing machine (UTM) and dynamic mechanical analysis (DMA), and the thermal properties were tested using differential scanning calorimetry (DSC) and thermogravimetric analysis (TGA). The experimental data were used to analyze the effects of the amount of added P-polyol on the properties of WPUs.

## 2. Experimental

### 2.1. Materials

PCD (number average molecular weight (M_n_) of 2000 g/mol) was obtained from Asahi Kasei Chemicals Corporation (Tokyo, Japan). IPDI (98% purity), 1-methyl-2-pyrolidone (NMP, 99% purity), DMPA (98% purity), dibutyltin dilaurate (DBTDL, 95% purity), triethylamine (TEA, 99.5% purity), and ethylene diamine (ED, 98.5% purity) were purchased from Sigma-Aldrich (St. Louis, MO, USA) and were used as received without further purification. Ethylene glycol and phenylphosphonic dichloride (PPD) were also obtained from Sigma-Aldrich (St. Louis, MO, USA). Acetone (99.5% purity) and methylene chloride (99.5% purity) were acquired from Samchun Chemicals (Seoul, Korea).

### 2.2. Synthesis of P-Polyol

P-polyol was synthesized using a previously reported method [22]. For a typical synthesis, trimethylamine (30 g), ethylene glycol (10 g), and methylene chloride (30 mL) were added to a 250 mL three-neck flask. A solution of PPD (27.5 g) in methylene chloride (50 mL) was added to the flask using a dropping funnel for 24 h at 25 °C. After filtering to separate the solids, the solution was washed three times with deionized water in a separation funnel and the filtrate was dried in an oven at 40 °C. The hydroxyl value of P-polyol was measured using the titration method, and the synthesized P-polyol was used without further purification. 

### 2.3. Synthesis of WPUs

The reaction scheme for the synthesis of WPUs is presented in Figure 1. PCD and P-polyol were added to a 500 mL three-neck flask equipped with a mechanical stirrer. The flask was stirred and heated to 80 °C until PCD dissolved completely. Thereafter, IPDI (26 mL) was injected into the flask, followed by the addition of DBTDL (0.8 μg), and the reactants were stirred for 10 min. Next, DMPA dissolved in NMP (20 mL) was added to the flask and the reactants were stirred for 20 min. After the temperature of the solution was lowered to 50 °C, acetone (77 g) was added. A solution of TEA (4.3 g in 30 mL of acetone) in acetone was subsequently added for 1 h. Deionized water (200 g) was slowly added for 40 min using a dropping funnel under stirring at 1000 rpm to prepare WPUs. The terminal –NCO groups of polyurethane were capped by adding a solution of ED (10 g) in water for 20 min. The reaction mixture was stirred for 1 h and stored in a wide-mouth bottle. The P-polyol:PCD molar ratio was adjusted from 0.1:0.9 to 0.2:0.8 and 0.3:0.7 to prepare PU-10, PU-20, and PU-30 WPUs, respectively, with different physical properties; in addition, a P-polyol–free WPU (hereinafter, Ref) was synthesized and used as a reference (Table 1).

### 2.4. Preparation of WPU Films 

The prepared WPUs (10 g) were poured into Petri dishes (90 mm of diameter) and dried for 15 h at 50 °C and 5 h at 70 °C in a dry oven to fabricate WPU films. The prepared films were used for mechanical and thermal analyses.

### 2.5. Characterization

The molecular weight of P-polyol was measured by determining its hydroxyl value, according to the ASTM E1899-08 method, using a Metrohm 888 Titrando (Metrohm AG, Herisau, Switzerland) instrument. The synthesis of WPUs was monitored using a Fourier-transform infrared spectroscopy (FT-IR; Nicolet 6700/Nicolet Continuum; Thermo Fisher Scientific Inc., Waltham, MA, USA) system. The molecular weight of WPU was measured using a liquid chromatography (1260 Infinity II, Agilent Technologies, Santa Clara, CA, USA) apparatus in tetrahydrofuran solvent with a constant flow rate of 1 mL/min. The particle size of WPUs was measured using a particle size analyzer (PSA; Mastersizer 3000, Malvern PANalytical, Malvern, UK) in deionized water. Dog bone test samples were prepared to evaluate the tensile strength of the films, which was measured five times using a UTM (Lloyd LF plus, TangentLABs, Indianapolis, IN, USA) and the ISO 37 Type-4 method. The load cell and crosshead speed of the UTM were 10 N and 100 mm/min, respectively. Kinetic data on the WPU films were collected using a DSC (Q2000, TA Instruments, New Castle, DE, USA) apparatus at a heating rate of 10℃/min and in the temperature range of −80 to 250 °C. The viscoelastic properties of the WPU films were tested using a DMA (Q800, TA Instruments, New Castle, DE, USA) unit and 60 mm × 12 mm × 3 mm samples at a frequency of 1 Hz with 10 μm oscillation amplitude and heating rate of 5 °C/min in the temperature range of −80 to 80 °C under a tension clamp. The thermal stability of the films was measured using a TGA (Q500, TA Instruments, New Castle, DE, USA) instrument in the temperature range of 25–800 °C at a heating rate of 10 °C/min under N_2_. 

## 3. Results and Discussion

### Characterization of WPUs

**Figure 2:**
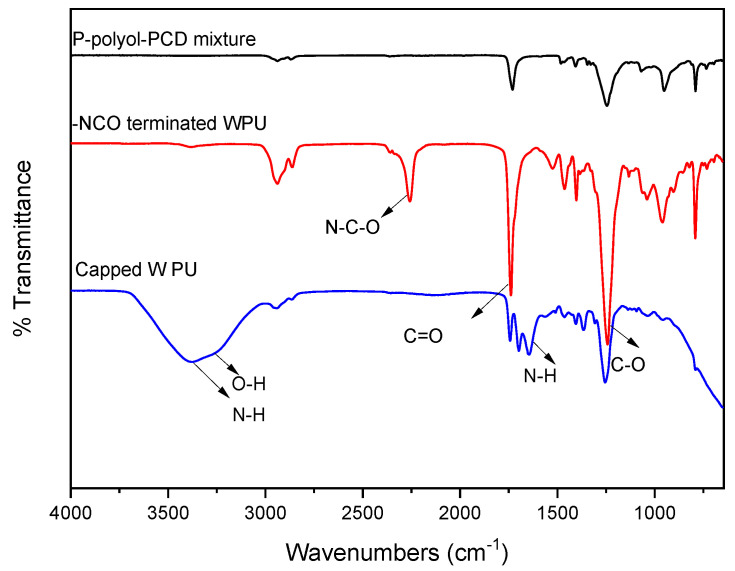
FT-IR data of WPU and polyol mixture.

The measured hydroxyl value of P-polyol was determined to be 62.3 mg KOH (the amount of KOH (in mg) to neutralize any acid) using the titration method. The molecular weight of P-polyol was calculated to be 450 g/mol by dividing 56.11 g/mol by the hydroxyl value (62.3 mg KOH/g) and the number of –OH groups (2) of P-polyol. The structure of the synthesized WPU samples was confirmed using FT-IR spectrometry (Figure 2). After IPDI was added to the P-polyol–PCD mixture, a typical –NCO peak at 2275 cm^−1^ emerged in the FT-IR spectrum. The terminal –NCO peak disappeared when ED, which served as the chain extender, was added to the reaction mixture (Figure 2). The characteristic N–H peaks of ED appeared at 3200 and 1660 cm^−1^, and the –OH peak of water was detected at 3400 cm^−1^ and was ascribed to the addition of water to prepare WPUs. 

The molecular weight of WPUs was measured using liquid chromatography, and the results are summarized in Table 2. The M_n_ values of the WPUs samples ranged between 17,540 and 20,095 g/mol.

**Figure 3:**
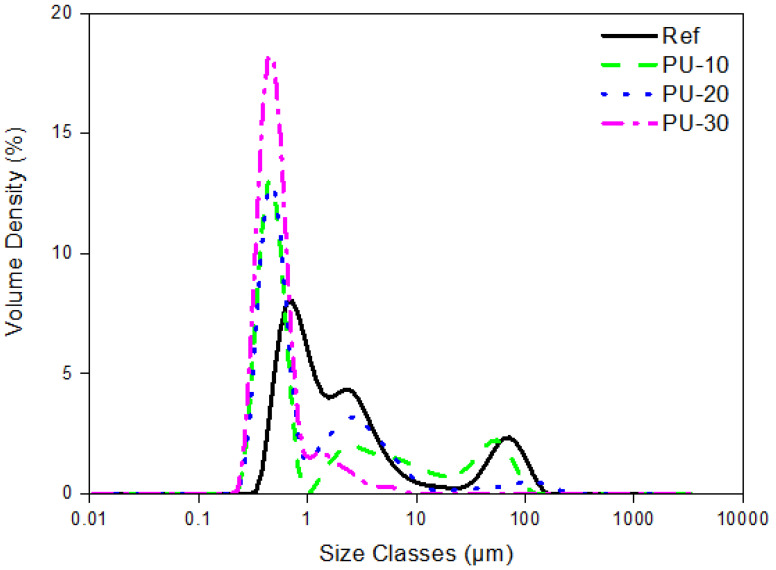
Particle size distribution of waterborne polyurethane (WPU) samples determined using a particle size analyzer. Here, Ref denotes P-polyol–free WPU and PU-10, PU-20, and PU-30 denote WPUs with P-polyol:PCD molar ratios of 0.1:0.9, 0.2:0.8, and 0.3:0.7, respectively.

The average size (Sauter mean diameter) of WPU particles in water was determined using the aforementioned PSA, and the results showing the trimodal distribution pattern are presented in Figure 3. The particle size of Ref was 1.21 μm, and it decreased to 0.70, 0.68, and 0.49 μm for the PU-10, PU-20 and PU-30 samples, respectively, with increasing P-polyol amount. It has been reported that smaller WPU particles are correlated with higher hydrophilicity [23,24]. The decrease in particle size with increasing P-polyol amount suggested that P-polyol presented hydrophilic properties. The hydrophilic properties of phosphate monomers were reported by Puri et al. [25], who indicated that the addition of ethylene glycol methacrylate phosphate to polymethyl methacrylate resin leads to hydrophilicity, increasing proportionally to the amount of phosphate monomer.

**Figure 4:**
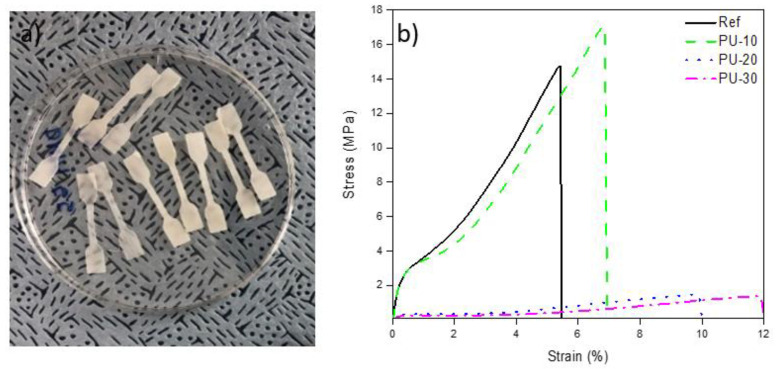
Tensile strength measurements: (**a**) Photographs of dog bone test samples and (**b**) stress–strain curves of waterborne polyurethane (WPU) samples. Here, Ref denotes P-polyol–free WPU and PU-10, PU-20, and PU-30 denote WPUs with P-polyol:PCD molar ratios of 0.1:0.9, 0.2:0.8, and 0.3:0.7, respectively.

Tensile strength measurements were performed to analyze the mechanical properties of the WPU film, and the results are illustrated in Figure 4. The addition of P-polyol increased the tensile strength by 16% (from 14.7 (±0.6) MPa for Ref to 17.1 (±0.2) MPa for PU-10). However, the tensile strength of the WPU films with higher P-polyol contents decreased abruptly.

**Figure 5:**
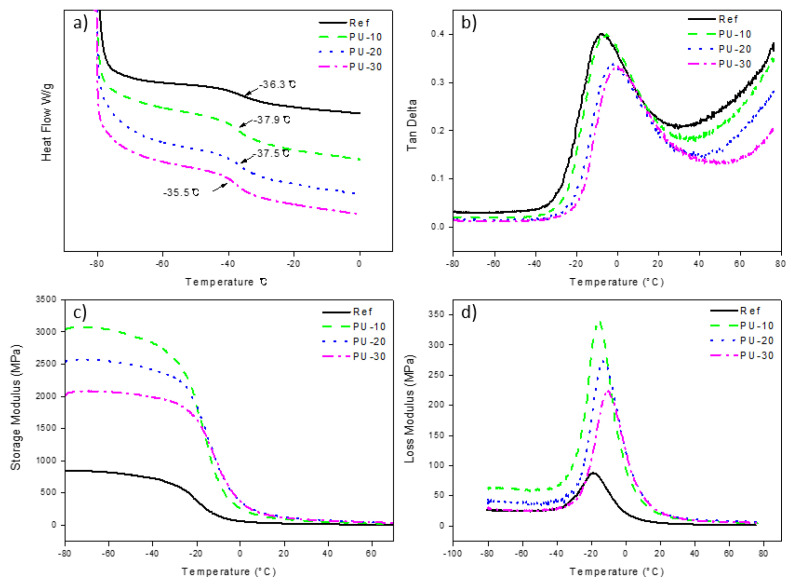
(**a**) Differential scanning calorimetry data obtained as averages of two runs. (**b**) Tan δ, (**c**) storage modulus, and (**d**) loss modulus of waterborne polyurethanes (WPUs). Here, Ref denotes P-polyol–free WPU and PU-10, PU-20, and PU-30 denote WPUs with P-polyol:PCD molar ratios of 0.1:0.9, 0.2:0.8, and 0.3:0.7, respectively.

Polyurethanes present two glass transition temperatures (T_g_), one for the soft segment and another for the hard segment. The T_g_ of the soft segment of Ref was determined to be −36.31 °C by averaging the results of two DSC runs (Figure 5a). The T_g_ values of the other analyzed WPUs were similar. However, the T_g_ values obtained using DMA (tan δ) were different depending on the amount of P-polyol (Figure 5b and Table 3). The T_g_ of PU-10 (−5.4 °C) was 2.6 °C higher than that of Ref (−8.0 °C). The difference in the T_g_ values of PU-20 (−3.0 °C) and PU-30 (−0.8 °C) was similar (2.2 °C). The addition of P-polyol to PCD caused the storage modulus of the WPU samples to change from 700 MPa for Ref to 3042 MPa for PU-10 (Figure 5c), owing to the rigid structure of P-polyol. However, when the amount of P-polyol was further increased, the storage modulus of the WPU samples decreased. The increase in loss modulus with increasing P-polyol amount followed a similar trend to T_g_ (Figure 5d).

**Figure 6:**
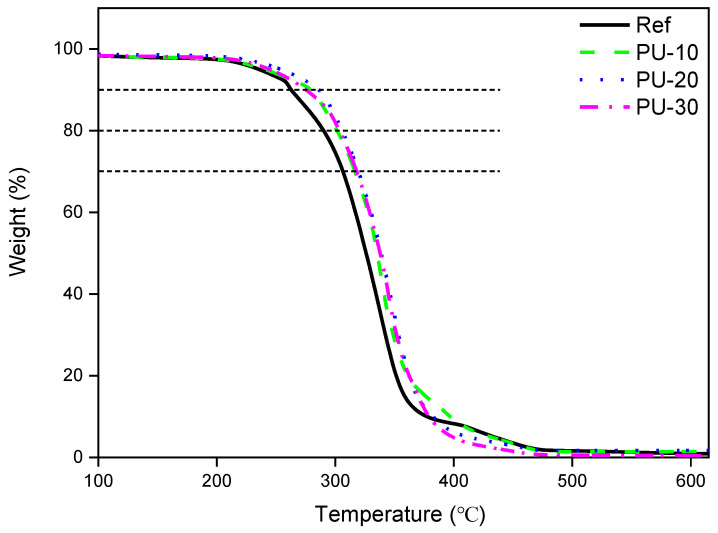
Thermogravimetric analysis curves of waterborne polyurethanes (WPUs). Here, Ref denotes P-polyol–free WPU and PU-10, PU-20, and PU-30 denote WPUs with P-polyol:PCD molar ratios of 0.1:0.9, 0.2:0.8, and 0.3:0.7, respectively.

The thermal stability of WPUs was tested using TGA, and the results are presented in Figure 6 and Table 4. The decomposition temperatures (T_d_) of Ref and PU-10 at 10% of weight loss were 252 and 279 °C, respectively, and T_d_ of PU-20 (286 °C) was 34 °C higher than that of Ref. This indicated that the addition of P-polyol to PCD enhanced the thermal stability of the WPU samples. The differences in T_d_ values at 20% and 30% of weight loss of all samples were on average 10 °C lower than the differences in T_d_ values at 10% and 20% of weight loss; however, the addition of P-polyol to PCD effectively improved the thermal stability of the analyzed WPU samples.

## 4. Conclusions

P-polyol, which contained phosphorous and a rigid aromatic group, was synthesized and subsequently used to fabricate WPUs. The particle size of the prepared WPUs decreased with increasing P-polyol content; however, the M_n_ values of the prepared WPU sampled were similar and ranged between 17,000 and 20,000 g/mol. Tensile testing revealed that the tensile strength of PU-10 (17 MPa) was higher than that of Ref (14 MPa). However, when the P-polyol content was increased to over 20%, the tensile strength decreased abruptly. DMA experiments indicated that T_g_ of the WPU samples increased by approximately 2 °C with increasing P-polyol content by 10%. Furthermore, the addition of P-polyol to PCD increased the thermal stability of the synthesized WPUs, leading to the T_d_ of PU-10 at 10% of weight loss, being 27 °C higher than that of Ref. Therefore, it was concluded that the addition of 10% P-polyol to PCD during the synthesis of WPU increased tensile strength and thermal decomposition temperature by 16% and 27 °C, respectively.

## Figures and Tables

**Table 1:** Composition of waterborne polyurethanes (WPUs); P-polyol–free WPU (Ref); PU-10, PU-20, and PU-30 denote WPUs with P-polyol:PCD molar ratios of 0.1:0.9, 0.2:0.8, and 0.3:0.7, respectively; phosphorus-containing polyol (P-polyol), polycarbonate diol (PCD), dimethylol propionic acid (DMPA), triethylamine (TEA), and ethylene diamine (ED).

Sample	PCD (g)	P-Polyol (g)	DMPA (g)	TEA (g)	ED (g)	Water (g)
Ref	75	0	5.7	4.30	2.49	200
PU-10	75	1.7	5.7	4.30	2.26	200
PU-20	75	3.4	5.7	4.30	2.03	200
PU-30	75	5.7	5.7	4.30	1.81	200

**Table 2:** Molecular weight of waterborne polyurethane (WPU) samples; here, M_n_, M_w_, and D denote the number average molecular weight, mass average molecular weight, and polydispersity, respectively; Ref denotes P-polyol–free WPU; and PU-10, PU-20, and PU-30 denote WPUs with P-polyol:PCD molar ratios of 0.1:0.9, 0.2:0.8, and 0.3:0.7, respectively.

Sample	M_n_ (g/mol)	M_w_ (g/mol)	Ð
Ref	17,540	37,427	2.13
PU-10	18,397	37,360	2.03
PU-20	20,095	40,778	2.03
PU-30	17,915	37,077	2.07

**Table 3:** Dynamic mechanical analysis data of waterborne polyurethanes (WPUs); here, Ref denotes P-polyol–free WPU and PU-10, PU-20, and PU-30 denote WPUs with P-polyol:PCD molar ratios of 0.1:0.9, 0.2:0.8, and 0.3:0.7, respectively.

Sample	Tan δ (°C)	Storage Modulus (MPa)	Loss Modulus (°C)
Ref	−8.0	832	−19.1
PU-10	−5.4	3042	−15.3
PU-20	−3.0	2532	−13.4
PU-30	−0.8	2019	−10.2

**Table 4:** Decomposition temperatures (T_d_) of waterborne polyurethanes (WPUs) at different weight losses determined using thermogravimetric analysis measurements; here, Ref denotes P-polyol–free WPU and PU-10, PU-20, and PU-30 denote WPUs with P-polyol:PCD molar ratios of 0.1:0.9, 0.2:0.8, and 0.3:0.7, respectively.

Sample	Ref	PU-10	PU-20	PU-30
T_d_ at 10% weight loss (°C)	252	279	286	275
T_d_ at 20% weight loss (°C)	290	301	305	303
T_d_ at 30% weight loss (°C)	325	335	338	337
Char residue at 615 °C	0.8	1.4	1.7	0.4

## Data Availability

The data presented in this study are available in article.
